# Exceptionally strong, stiff and hard hybrid material based on an elastomer and isotropically shaped ceramic nanoparticles

**DOI:** 10.1038/s41598-017-07521-0

**Published:** 2017-08-04

**Authors:** Prokopios Georgopanos, Gerold A. Schneider, Axel Dreyer, Ulrich A. Handge, Volkan Filiz, Artur Feld, Ezgi D. Yilmaz, Tobias Krekeler, Martin Ritter, Horst Weller, Volker Abetz

**Affiliations:** 1 Institute of Polymer Research, Helmholtz-Zentrum Geesthacht, Max-Planck-Strasse 1, D-21502 Geesthacht, Germany; 2 Institute of Advanced Ceramics, Hamburg University of Technology, Denickestrasse 15, D-21073 Hamburg, Germany; 3 Institute of Physical Chemistry, Hamburg University, Martin-Luther-King-Platz 6, D-20146 Hamburg, Germany; 4 Electron Microscopy Unit, Hamburg University of Technology, Eißendorferstraße 42, D-21073 Hamburg, Germany

## Abstract

In this work the fabrication of hard, stiff and strong nanocomposites based on polybutadiene and iron oxide nanoparticles is presented. The nanocomposites are fabricated via a general concept for mechanically superior nanocomposites not based on the brick and mortar structure, thus on globular nanoparticles with nanosized organic shells. For the fabrication of the composites oleic acid functionalized iron oxide nanoparticles are decorated via ligand exchange with an α,ω-polybutadiene dicarboxylic acid. The functionalized particles were processed at 145 °C. Since polybutadiene contains double bonds the nanocomposites obtained a crosslinked structure which was enhanced by the presence of oxygen or sulfur. It was found that the crosslinking and filler percolation yields high elastic moduli of approximately 12–20 GPa and hardness of 15–18 GPa, although the polymer volume fraction is up to 40%. We attribute our results to a catalytically enhanced crosslinking reaction of the polymer chains induced by oxygen or sulfur and to the microstructure of the nanocomposite.

## Introduction

Inspired by biomaterials like nacre the combination of high aspect ratio ceramic nanoparticles and a soft organic matrix is commonly accepted as the pathway to stiff, strong and damage tolerant nanocomposites^1^. Using for instance clay-nanoparticles with an aspect ratio *r* ≈ 50–1000^2^ the tensile stresses *σ*_*n*_ in the nanoparticles are downscaled to low shear stresses ${\tau }_{P}\propto \frac{{\sigma }_{n}}{r}$, which can be carried by the mechanically weak organic matrix. As the length and width of the ceramic nanoparticles is typically smaller than 100 nm these particles are flaw tolerant and, therefore, very strong due to their covalent/ionic bonds^1^. This mechanical concept provides likewise hard, stiff and strong as well as damage tolerant nanocomposites, which overcome the weakness of one material class and achieve multi-functionality^2–7^.

Recently, it was shown that an FCC-superstructure of self-assembled monodisperse spherical iron oxide nanoparticles with oleic acid shells leads to a very hard, stiff and strong nanocomposite^8^. Moderate heat treatment up to 350 °C triggered the cross-linking of the oleic acid molecules, which were attached to the iron oxide by their carboxyl groups and resulted in nanocomposites with a nanohardness of 4 GPa, an elastic modulus of 80 GPa and a strength of 500 MPa in micromechanical bending tests^8^. This result demonstrates that the above mentioned concept based on high aspect ratio nanoparticles is not necessary to achieve hard, stiff and strong nanocomposites^8^. Globular nanoparticles with a very thin organic shell (thickness of the shell smaller than 5 nm) may also lead to excellent mechanical properties, which is realized by the aragonite platelets of nacre^9^. We assume that stiffening of the organic phase caused by crosslinking and a confinement effect, which reduces the entropic freedom of the organic phase are the main reasons for these mechanical properties. The objective of this work is to show that this concept can be applied to other iron oxide nanocomposites, where the oleic acid is replaced by α,ω-polybutadiene dicarboxylic acid, and the overall processing temperature is reduced to 145 °C.

## Results and Discussion

**Figure 1:**
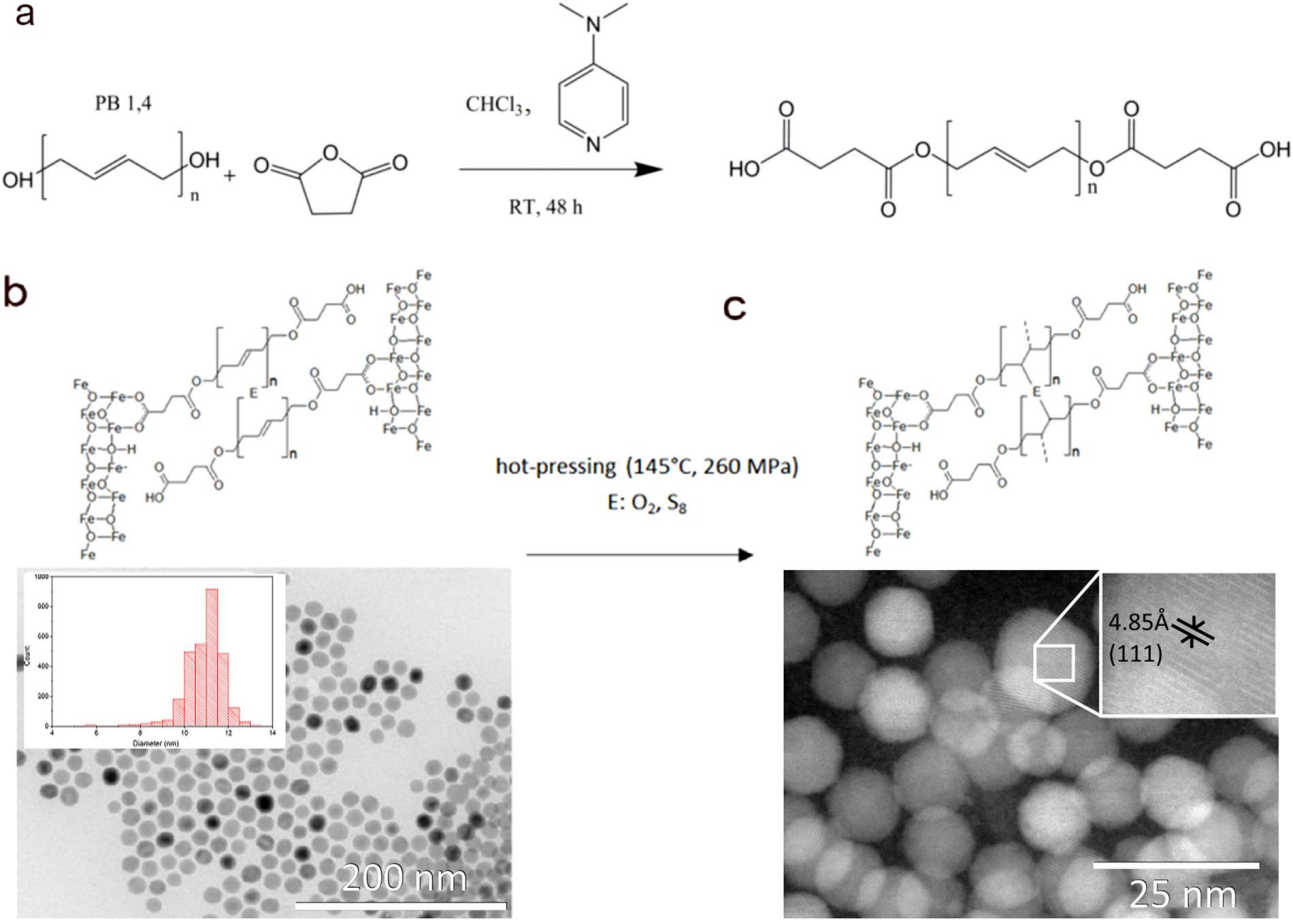
(**a**) End-functionalization of polybutadiene with carboxyl end-functional groups (conversion of α,ω−polybutadiene diol into α,ω-polybutadiene dicarboxylic acid). (**b**) TEM micrographs of the primary 11 nm-sized iron oxide nanoparticles with their corresponding size distribution histogram (insets) and a scheme of their inter-particular spaces. (**c**) After particle sedimentation, drying and hot pressing black pellets of the iron oxide/α,ω-polybutadiene dicarboxylic acid/sulfur nanocomposites were obtained and investigated by STEM. The particles exhibit no major change after hot pressing like faceting or sintering. The highlighted line spacing of 4.85 Å correspond to the (111) lattice plane distance of Fe_3_O_4_.

We used the commercially available low molecular weight Poly bd® R-45HTLO hydroxyl terminated polybutadiene resin which we chemically modified in order to get carboxyl functional end groups^10, 11^. This type of polybutadiene (PB) can be correlated to the oleic acid used in a previous work since it has low number average molecular weight (2800 g/mol) and additionally double bonds which could be used for crosslinking. The end-functionalized polymer was precipitated in cold methanol and washed thoroughly with distilled water. The reaction scheme is presented in Fig. 1(a). PB was chemically characterized by nuclear magnetic resonance spectroscopy (^1^H-NMR), Fourier transform infrared spectroscopy (FT-IR) and differential scanning calorimetry (DSC). Details on the polymer characterization are given in chapter A of the Supporting Information (SI).

The iron oxide nanoparticles were synthesized according to Yu *et al*.^12^. Hereby, particles with an average diameter of 10.9 ± 0.9 nm were synthesized. The average diameter was determined by a statistical analysis of several hundreds of particles acquired through transmission electron microscopy (TEM) investigations (Fig. 1(b)). Selected area electron diffraction (SAED) and X-ray diffraction (XRD) validated the magnetite structure of their inorganic core^12^. The organic shell was identified as oleic acid by attenuated total reflectance infrared spectroscopy (ATR-IR). The organic fraction of the particles was measured by thermogravimetric analysis (TGA). Details of the particle synthesis and characterization are given in SI, chapter B.

A mixture of oleic acid coated magnetite particles and dissolved PB in tetrahydrofuran (THF) was used to exchange the oleic acid by PB. The exchange mechanism of different ligands is described in literature^13–17^ and can be explained by thermodynamic considerations (minimum of Gibbs free energy). Assuming that the PB binds on the particles with the same binding energy as the oleic acid and taking into account the difference in the size of oleic acid and PB, the use of excess PB (ratio of 1:400 of oleic acid/PB) leads to the exchange of more than one oleic acid molecules on the particles. This exchange of some oleic acid molecules with PB chains implies that the enthalpy difference Δ*H* after and before the exchange is positive. Because of the relatively large gain in translational entropy by the free oleic acid molecules after the ligand exchange and an only limited loss of conformational entropy of the PB chains which are attached to the nanoparticles, the entropy term (−*T*Δ*S*) still overcompensates the positive enthalpy difference (Δ*H*), leading to a negative difference of the Gibbs free energy (Δ*G* = Δ*H* − *T*Δ*S*) before and after the exchange. However, a small amount of oleic acid is expected to remain on the surface of the nanoparticles; thus this is very small, and the formation of an additional layer of PB cannot occur. ATR-IR on both particle systems clearly indicates a successful exchange of the ligand (SI chapter B, Figure SI 2). TGA proves mass fractions for PB on the 11 nm-diameter magnetite particles of 16.4 ± 0.1 weight-%.

To obtain a solid bulk material, the PB coated iron oxide nanoparticles were dispersed in THF and poured into a sealed die. The aggregation of the nanoparticles was induced by slow evaporation of solvent within several days at room temperature. Thereafter, the remaining sediment was dried under vacuum at room temperature and uniaxially pressed in air at 145 ± 5 °C with a pressure of 260 MPa for 7 hours.

To crosslink the PB coated particles by sulfur (vulcanization), 493 mg of these particles were dispersed in 3 mL of a 0.74 weight-% THF sulfur solution. This corresponds to a content of 15.11 weight-% sulfur related to the PB fraction. According to the preparation described before the particles were sedimented, dried and pressed.

This procedure leads to pellets of a compact material consisting of disordered nanoparticles (Fig. 1(c)). The obtained materials consisting of iron oxide/PB nanocomposites without and with sulfur have Archimedean densities of 2.42 g ∙ cm^−3^ and 2.73 g ∙ cm^−3^, respectively. Combined with the corresponding helium pycnometric particle densities of 2.4552 ± 0.0090 g ∙ cm^−3^ and 2.8275 ± 0.0090 g ∙ cm^−3^, the open porosities were calculated and they are approximately 1.64% for the PB and 3.48% for the vulcanized PB iron oxide nanocomposites, respectively (Table 1).

**Table 1:** Summary of physical properties of the Fe_3_O_4_/PB nanocomposites.

	ρ_A_ g ∙ cm^−^³	ρ_He_ g ∙ cm^−^³	P %	f_m,poly_ weight-%	E_NI_ GPa	H_NI_ GPa	E_B_ GPa	σ_B_ MPa	E_C_ GPa	σ_C_ GPa
Fe_3_O_4_@PB	2.42	2.45	1.6	16.4	10.1 ± 0.4	0.21 ± 0.04	20 ± 5	191 ± 10	14 ± 1	369 ± 111
Fe_3_O_4_@PB + S	2.73	2.83	3.5	16.4	21.2 ± 1.5	0.83 ± 0.12	23 ± 8	184 ± 87	8 ± 2	476 ± 255

ρ_A_: Archimedian density, ρ_He_: He-pycnometric density, f_m,poly_: mass fraction of polymer, E: elastic modulus measured by nanoindentation (NI), microbending (B) and microcompression (C) and σ: fracture strength of microbending (B) and microcompression (C).

The iron oxide/PB with sulfur nanocomposite was additionally investigated by CHNS-elemental analysis and energy-dispersive X-ray analysis (EDX). From the CHNS elemental analysis the mass fractions of carbon, hydrogen, nitrogen, and sulfur in the nanocomposites were determined with 12.67 weight-%, 0.89 weight-%, 0.87 weight-% and 2.56 weight-%, respectively. This corresponds to a sulfur content in the organic phase of 15.06 weight-%. In comparison with the elemental analysis EDX detects an average of 3.43 ± 0.82 weight-% sulfur in the nanocomposite, which corresponds to 17.0 ± 4.0 weight-% in the organic phase. This is in line with the value obtained from the elemental analysis and the desired composition. Details are given in SI, chapter B.

The sulfur content is thus comparable to ebonite (hard rubber)^18^. The molar ratio between sulfur atoms and repeating units of PB is approximately 1:3. Assuming the formation of sulfur bridges with two sulfur atoms, these crosslinks occur on average at every sixth repeating PB unit. Additional crosslinks to the ones caused by sulfur increase further the crosslink density, leading to a mechanically rigid organic phase.

The mechanical properties of the nanocomposites were investigated by nanoindentation, micromechanical bending, and micromechanical compression tests, because the macroscopic integrity of the samples was not suitable for macroscopic bending tests. For the nanoindentation tests, the system is equipped with a Berkovich tip. The summarized results from an array of 5 × 5 indentations with a maximum depth of 2000 nm are given in Table 1 and show a 2 times higher modulus and 4 times higher hardness for the vulcanized nanocomposites. For a polymer weight fraction of 16% corresponding to a volume fraction around 45% the measured nanohardness of 0.8 GPa and elastic modulus of 21 GPa belong to the highest reported values^8, 19^.

Micromechanical tests were performed with micrometer-sized triangular shaped cantilevers for microbending and cylindrical pillars for microcompression, both isolated from the bulk using a focused ion beam (FIB). The microbending and microcompression tests were conducted in the nanoindenter equipped with a Berkovich tip and a flat-ended punch the latter being 10 µm in diameter, respectively. Force, displacement, and stiffness data were recorded continuously as a function of indentation displacement.

**Figure 2:**
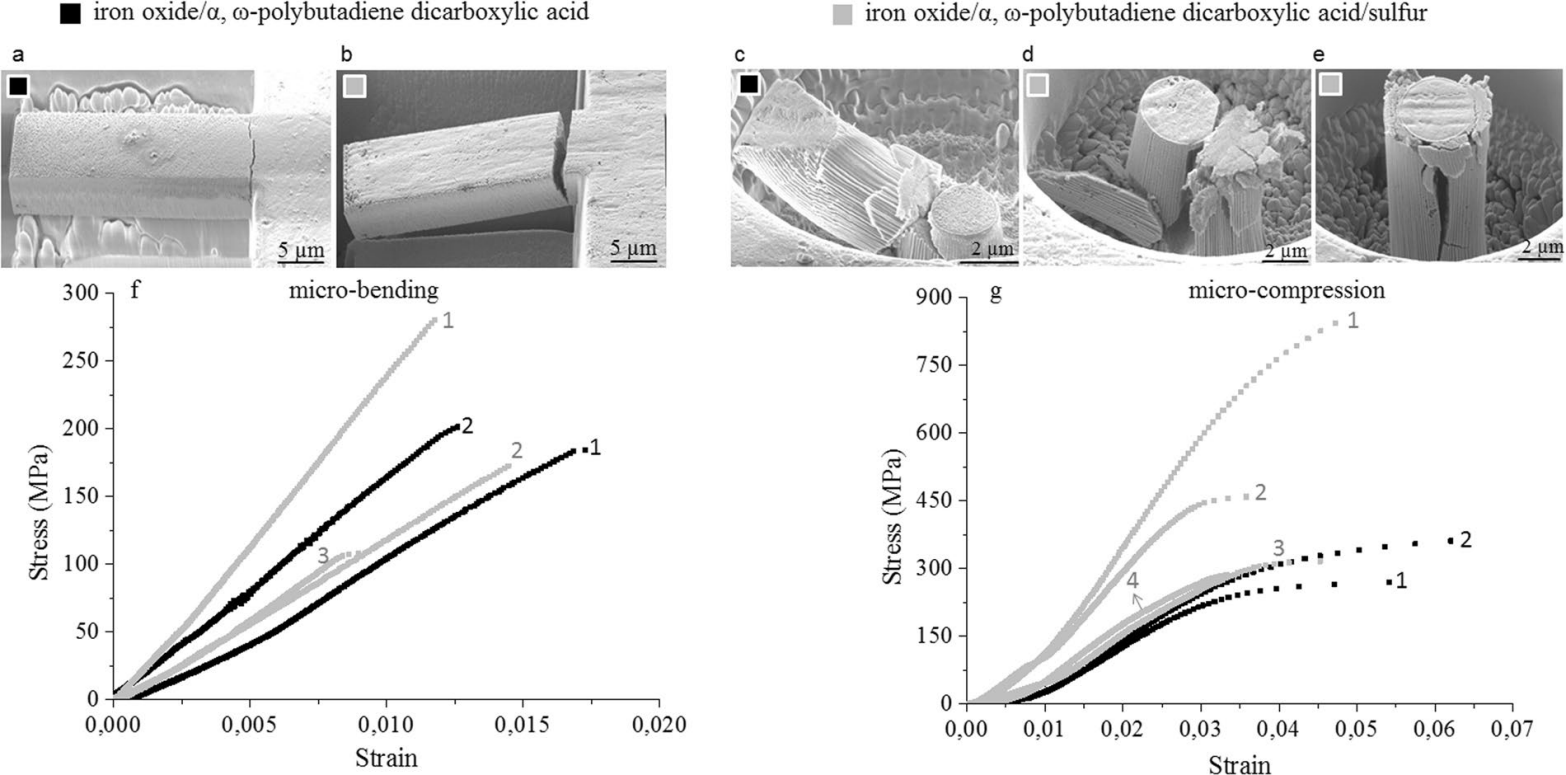
Micromechanical test of the nanocomposites. (**a**–**e**) Representative failure modes of the nanocomposite iron oxide/α,ω-polybutadiene dicarboxylic acid (black, **a** and **c**) and iron oxide/α,ω-polybutadiene dicarboxylic acid/sulfur (grey, (**b**,**d**,**e**)). Stress-strain curves obtained under micro-bending (**f**) and micro-compression (**g**) are plotted. Specimen legends 1–4 are given in Tables S2 and S3 in supplementary information.

The bending and compression failure modes are illustrated in Fig. 2(a–e). All micro-cantilevers revealed linear elastic brittle failure (Fig. 2(f)) with large crack splitting in the cantilevers from the bulk (Fig. 2(a and b)). The compression specimens exhibited different failure modes such as shearing and buckling (Fig. 2(c)), end crushing (Fig. 2(d) and axial splitting (Fig. 2(e)). These differences in the failure modes are probably the reason for the large scatter in the resulting failure strengths.

The resultant stress-strain curves of all specimens are presented in Fig. 2(f and g). In contrast to the bending tests, the compression specimens showed a large non-linear region before fracture. The microbending and microcompression failure strength and elastic modulus results are listed in Table 1. More details of the micromechanical tests are given in SI, chapter C according to the literature^20, 21^. Besides our own results on oleic acid/iron oxide nanocomposites with a more than two times lower organic content, the micromechanical tests show the highest bending strength in the range of 100–300 MPa and compressive strengths between 300–900 MPa ever reported for hard nanocomposites.

In order to elucidate the crosslinking reaction, the reaction kinetics and the resulting entanglement molecular weight of PB with and without sulfur were investigated via shear rheological experiments in the oscillatory mode. The polymer was placed between two parallel plates with a diameter of 25 mm and a distance of 0.2 mm, while an oscillatory shear deformation with a constant shear amplitude of 5% and an angular frequency of 0.1 rad/s was applied. All measurements were performed at 145 °C under air and nitrogen atmosphere, respectively, to verify the influence of oxygen on the crosslinking reactions at the processing temperature. Under these conditions no significant influence of hydrogen bonds between the carboxylic groups^22, 23^ on the rheological properties is expected. As it is known that anhydride can only be formed at significantly higher temperatures above 200 °C as it is referred in works on polyimide and polymers of intrinsic microporosity^24, 25^, we can also rule out a possible increase of molecular weight of PB by polycondensation. Details of the rheological measurements are given in the SI chapter D.

**Figure 3:**
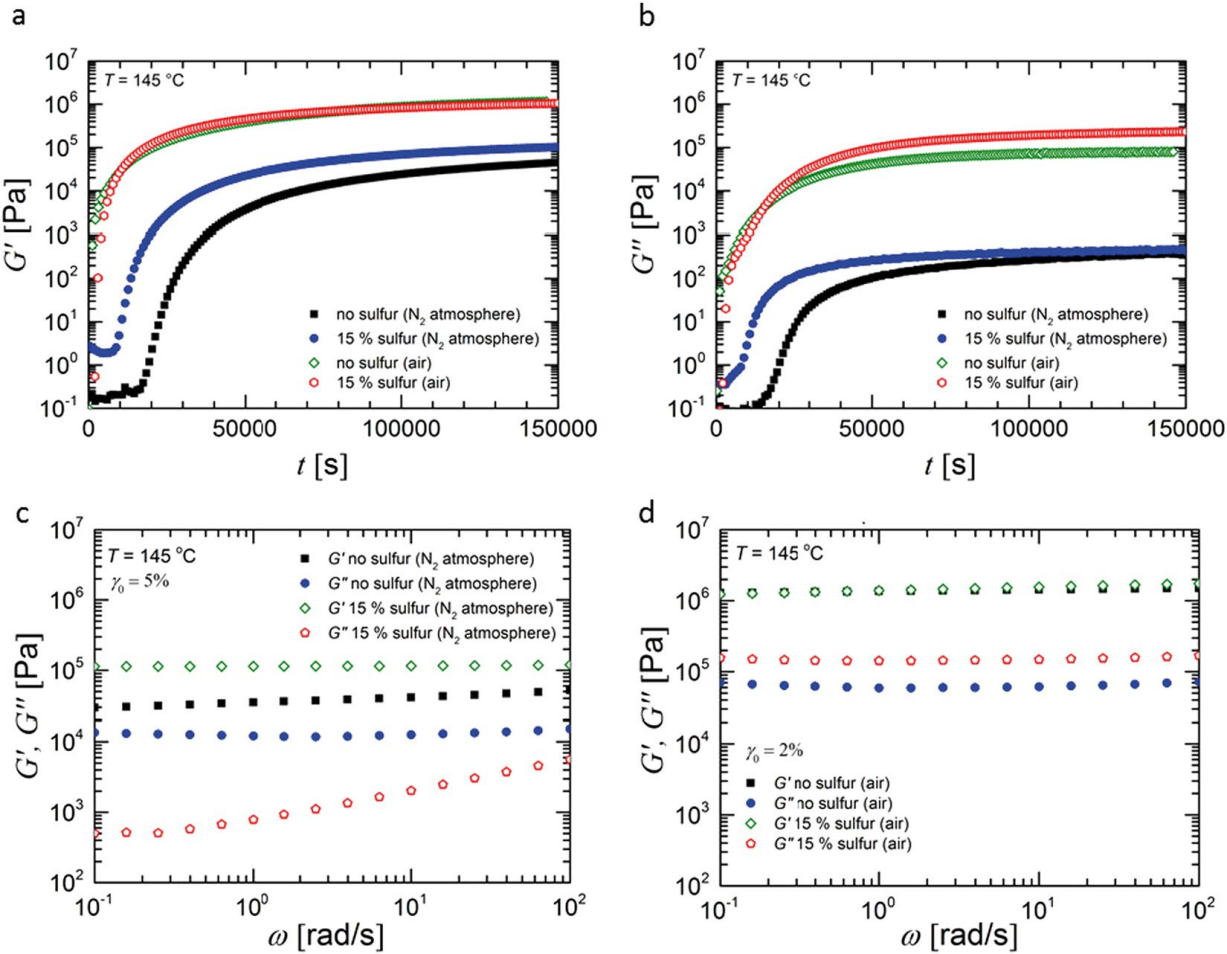
(**a**) Storage modulus *G′* and (**b**) loss modulus *G″* of α,ω-polybutadiene dicarboxylic acid as a function of time *t* during the thermal treatment at 145 °C. The time-dependent increase of the moduli indicates an oxygen induced polymerization and an oxygen supported vulcanization of α,ω-polybutadiene dicarboxylic acid. The angular frequency was *ω* = 0.1 rad ∙ s^−1^ and the shear amplitude *γ*_0_ = 5%. The storage modulus *G′* increases most rapidly for the crosslinking reactions in air. Results of frequency sweep experiments after the vulcanization reaction of α,ω-polybutadiene dicarboxylic acid in (**c**) a nitrogen atmosphere and (**d**) an air atmosphere. The graphs present the dynamic moduli *G′* and *G″* as a function of angular frequency ω.

The time sweep experiments yield the time-dependent dynamic moduli *G′* and *G″* during crosslinking of PB with and without addition of sulfur. Figure 3(a and b) reveal that the dynamic moduli of PB with and without sulfur increase by approximately 4 orders of magnitude within 1 day. In a nitrogen atmosphere, the increase of *G*′ for PB is faster in the case with sulfur than in the case without sulfur. An indication of a plateau for *G*′ was only found for the sulfur containing PB at approximately 30 h with a characteristic vulcanization time *t*_90_ of 28 h. However, the data in Fig. 3(a and b) measured in air for PB with and without sulfur indicate an increase of the dynamic moduli to much higher values. Furthermore, crosslinking of the polybutadiene chains proceeds much faster in the presence of oxygen than in a nitrogen atmosphere. The presence of oxygen leads to a higher crosslink density since oxygen at elevated temperatures can react with the double bonds of PB (formation of radicals) leading to carbon-carbon crosslinks in addition to vulcanization via sulfur.

The storage modulus *G*′_Plateau_ at the minimum of the loss tangent of the sample without sulfur in air is approximately 1.6 ∙ 10^6^ Pa which is even higher than the plateau modulus *G*′_Plateau_ = 1.1 ∙ 10^6^ Pa of 1,4-polybutadiene^26^. Therefore, a high crosslinking density is achieved in air. Using the relation *M*_c_ = 4*ρ*_*PB*_ *RT*/(5 *G*′_Plateau_) with the density of the polymer *ρ*_*PB*_ = 0.9 g ∙ mL^−1^, the measurement temperature *T* = 418.15 K (145 °C), the universal gas constant *R* = 8.314 J ∙ mol^−1^ ∙ K^−1^, the crosslinking molecular weight is *M*_c_ = 1490 g ∙ mol^−1^ for the PB crosslinked under air and *M*_c_ = 2000 g ∙ mol^−1^ for the PB with sulfur crosslinked under air. Compared with the molecular weight of the pristine PB *M*_PB_ = 2800 g ∙ mol^−1^ we conclude that roughly every PB chain is crosslinked by 1 or 2 sulfur bonds or by 1 carbon-carbon bond for the cases of crosslinking with sulfur or under air, respectively. Assuming a completely crosslinked sample, the crosslinking molecular weight is given by *M*_*c*_ = *4ρ*_*PB*_ *RT/(5G*′_*Plateau*_ *)* which yields *M*_*c*_ = 48200 g ∙ mol^−1^ without sulfur under nitrogen and *M*_*c*_ = 22450 g ∙ mol^−1^ with sulfur under nitrogen. The comparison of these two calculated *M*_*c*_ values shows that sulfur leads to crosslinking of polybutadiene. However, these crosslinking molecular weights are obviously higher than the molecular weight of PB and can thus be treated only as apparent crosslinking molecular weights. We attribute this result to an incomplete crosslinking reaction under nitrogen atmosphere which possibly only leads to a fraction of crosslinked PB chains and a non-zero fraction of uncrosslinked polybutadiene chains, such that the samples after treatment under nitrogen consist of crosslinked PB which is “diluted” with uncrosslinked or cyclized PB chains^27^. This dilution effect causes a lower plateau modulus than a fully crosslinked sample and consequently (because of the relation *M*_*c*_ = *4ρ*_*PB*_ *RT/(5G*′_*Plateau*_ *))* a larger apparent crosslinking molecular weight. In the case of a “diluted” sample, the relation *M*_*c*_ = *4ρ*_*PB*_ *RT/(5G*′_*Plateau*_ *)* is not applicable anymore, since its derivation in the framework of the classical theory of elasticity (see, e.g., ref. 28) is based on the assumption that all polybutadiene chains are crosslinked (leading to the factor *ρ*_*PB*_). This assumption is not fulfilled for an incompletely crosslinked sample leading to a lower value of crosslinked chains per volume element. The crosslinking density can be estimated using the relation *G*′_Plateau_ = 2*cRT* with the number *n* of chains per volume and the number *c* of crosslinks per volume. For the PB crosslinked under air without sulfur the crosslinking density is *c* = 0.230 ∙ 10^−3^ mol ∙ cm^−3^ and *c* = 0.160 ∙ 10^−3^ mol ∙ cm^−3^ with sulfur. Under nitrogen we have *c* = 0.007 ∙ 10^−3^ mol ∙ cm^−3^ (without sulfur) and *c* = 0.014 ∙ 10^−3^ mol ∙ cm^−3^ (with sulfur). Thermal crosslinking of polybutadiene at higher temperatures via carbon-carbon crosslinking can be more favorable in presence of free-radicals (e.g. oxygen) than vulcanization with sulfur^29^, which is supported by our data showing a higher crosslink density in case of PB crosslinked without sulfur under air.

However, the reaction conditions (or crosslinking kinetics) without the presence of the iron oxide nanoparticles may be quite different from the ones in the nanocomposite, leading to a significantly higher crosslink density of the organic phase in the nanocomposite. Hence one can assume that the rheological experiments on bulk samples strongly underestimate the crosslink density in the nanocomposite. A catalytically enhanced full conversion of sulfur into crosslinks could lead to an organic phase with comparable mechanical properties as found for ebonite which typically has a Young’s modulus in the range of 2.0 to 2.5 GPa^30^.

For the discussion of the mechanical properties it is decisive whether all nanoparticles are completely surrounded by a uniform, stiff polymer phase (which forms a matrix) or whether there exists a percolating network of touching nanoparticles (which are directly connected without any polymer layer in between them). Recently, studies on the temperature dependence of highly filled poly(vinyl butyral)/alumina (PVB/α-Al_2_O_3_) or poly(vinyl butyral)/titanium dioxide (PVB/TiO_2_) composites revealed that both direct particle contacts and polymer-filler interactions may contribute to the mechanical response of the composites^31, 32^. To discuss the mechanical implications of the two microstructures with a completely uniform polymer shell and a percolation network, respectively, we start by modeling the microstructure where the nanoparticles are surrounded by a polymer matrix by a primitive cubic lattice with cubic grains. For the geometrical dimensions of this microstructure we introduce the volume fractions of the polymer *f*_*V*,*poly*_ and nanoparticles *f*_*V*,*grains*_ with *f*_*V*,*grains*_ + *f*_*V*,*poly*_ = 1. With the measured weight fractions of the organic phase *f*_*m*,*poly*_ given in Table 1 the corresponding volume fractions are calculated according to ${f}_{V,poly}=\frac{\rho }{{\rho }_{poly}}{f}_{m,poly}$ (*ρ*_*poly*_ density of the polymer phase, *ρ* density of the composite). For both the vulcanized and non-vulcanized polybutadiene compositions the volume fractions range between 40–45%. As the porosity is smaller than 4% for both compositions we neglect it for the purpose of this simple calculation.

**Figure 4:**
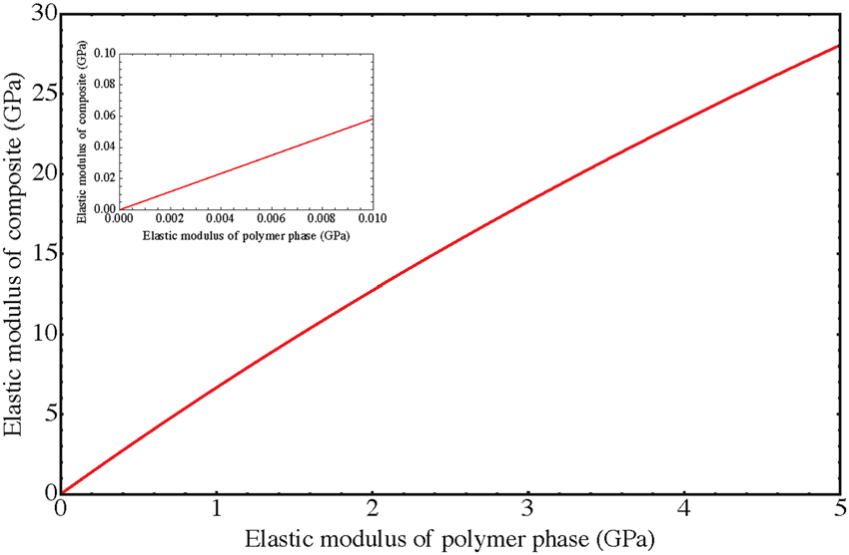
Elastic modulus of the nanocomposite *E*_*Comp*_ as a function of the elastic modulus of the polymer phase *E*_*P*_ calculated for a primitive lattice of cubic nanograins of size *g* = 8.8 nm and thickness of polymer matrix phase *p* = 1.8 nm. To achieve composite moduli on the order of 10–20 GPa the modulus of the polymer phase must be on the order of 2–4 GPa. The inset shows the modulus range around 1 MPa for the polymer, which was measured in the rheological experiments and leads to composite moduli which are 3 orders of magnitude lower than the measured ones.

To estimate the distances between the nanoparticles we introduce the edge length *g* of the cubic grains and the thickness *p* of the polymer sheath. Hence the unit cell of this primitive cubic lattice has the volume (*g* + *p*)^3^ and the relative organic sheath thickness is $\frac{p}{g}=\frac{1}{{(1-{f}_{V,poly})}^{1/3}}-1$. For a PB volume fraction in the order of 40% a relative sheath thickness *p*/*g* of approximately 0.2 is determined. The equivalent edge length *g* of a cube for a sphere with diameter *D* is ${(\frac{\pi }{6})}^{1/3}D\cong 0.81D$. With the nanoparticle diameter of ≈11 nm we get a corresponding average cubic grain size *g* of ≈8.8 nm and an average distance *p* between the cubic nanoparticles of ≈1.8 nm, which is filled by PB either vulcanized or not. Applying a 3D model of parallel and perpendicularly arranged cubic nanoparticles in a primitive cubic lattice with a polymer matrix leads to a composite modulus

1 $${E}_{Comp}={(\frac{\frac{p}{p+g}}{{E}_{p}}+\frac{\frac{g}{p+g}}{{E}_{//2}})}^{-1}$$

with ${E}_{//2}=\frac{p}{p+g}{E}_{P}+\frac{g}{p+g}{E}_{//1}$ and ${E}_{//1}=\frac{p}{p+g}{E}_{P}+\frac{g}{p+g}{E}_{g}$, and the elastic modulus of the nanoparticle and polymer *E*_*g*_ and *E*_*P*_, respectively. For the elastic modulus of the nanoparticle the value for magnetite *E*_*g*_ = 163 GPa is used^33^. Figure 4 shows a plot of the composite modulus *E*_*Comp*_ as a function of the elastic modulus of the polymer *E*_*P*_. It predicts a polymer modulus in the order of 2 to 4 GPa, which is in agreement with the values found for the catalytically enhanced crosslinked hard rubbers^30^. This modulus is approximately 1000 higher than the measured bulk modulus of the used PB in the rheological experiments. Therefore we conclude that it is most likely that the crosslink density in the nanocomposite is much higher than in the absence of the iron oxide nanoparticles.

On the other hand, if we assume a microstructure of a percolation network of directly touching nanoparticles this might explain the measured elastic moduli under compression as follows. If the volume fraction *f*_*V*,*perc*_ of load-bearing percolated columns is assumed parallel to the loading direction and the remaining fraction *f*_*V*,*non-perc*_ is the non-percolated volume fraction together with the polymer the composite modulus is ${E}_{Comp}={f}_{V,perc}{E}_{g}+{f}_{V,non-perc}{E}_{non-per}$. The non-percolated part will have a modulus of the order of some MPa because it will behave similar to the model given by equation (1) and is negligible. Hence with the measured composite moduli under compression the volume fraction of percolated touching nanoparticles should be approximately 5–12%. Indeed, the rheological experiments of Handge *et al*.^31^ showed that in the temperature/frequency range, in which the polymer phase behaves rubbery, the elastic modulus of the composite can attain values in the order of 1 GPa. Besides the microcompression tests we determined the elastic modulus also under bending, which produces tensile stresses at the loading side and compressive stresses on the other side. We emphasize that also under tensile stresses the percolation model of touching nanograins which are surrounded by a polymeric matrix may explain a high elastic modulus. The reason is the deformation of the matrix perpendicular to the loading direction, which results in a compressive stress and strain applied on the percolated three-dimensional network of nanograins. This compressive deformation perpendicular to the loading direction yields high stresses because of the percolated, load bearing paths perpendicular to the tensile direction. As the volume of the crosslinked PB in the elastic regime is almost constant^34^, the geometrical constraint of the percolated nanoparticles hinders the extension of the nanocomposite and qualitatively explains the measured high elastic modulus also in bending. Whether this model is also able to explain the high microbending strength of the nanocomposite is not clear.

Finally, we comment on a possible shift of the glass transition temperature to a value above room temperature, which would also explain the measured high elastic modulus. Such a shift of the glass transition temperature might be caused by the geometrical confinement or the crosslinking reaction itself as refereed in literature^35^. Therefore, we consider the geometrical confinement of the polymer within the approximately 1.8 nm wide gaps between the nanoparticles. The upper bound of a monolayer of PB is the root mean square end-to-end distance (Flory radius) ${R}_{F}=\sqrt{\langle {R}^{2}\rangle }$. For the used PB with a molar mass *M* = 2800 g ∙ mol^−1^ the Flory radius is 4.7 nm using the relation 〈*R* ^*2*^〉/*M* ≈ *0*.*8* Å^2^/(g mol^−1^)^36^ and results in a radius of gyration of *R*_*g*_ ≈ 1.9 nm. Hence, to fit within an average nanograin spacing of 1.8 nm the polymer is compressed in one dimension by 53% compared to its unperturbed state. Thus the tethered PB polymer chain is strongly deformed in the confined geometry.

This argument is also supported by considering the (hypothetical) radial position 〈*x*〉_N_ of the non-tethered end of the PB chain which is approximately given at a radial distance of 5.9 nm from the surface of the nanograin and thus would superimpose with the neighboured nanograin, estimated from the equation 〈*x*〉_N_ ≈ 1.25 nm·√Ν (*N* is the number of Kuhn segments of Kuhn length ≈1 nm^36^), as referred in literature^37^. Consequently, an entangled monolayer of PB bridges the gap between two neighboured iron oxide nanograins, where the fraction of chain segments having contact to the nanoparticles can be estimated by the ratio of the volume of a layer of thickness *k p* to the polymer volume. Depending on the layer thickness *k p* one finds for the volume fraction of monomers being in contact with the nanoparticles 9 *k p*/*D*, e.g., 17% for *k* = 1/9. Future investigations need to clarify whether this value is large enough in order to induce significant changes of the glass transition temperature.

In order to estimate the surface density of bound carboxyl groups to the iron oxide nanoparticles we calculate the number density of PB polymers as ${n}_{PB}=\frac{{\rho }_{PB}{N}_{A}}{{M}_{PB}}$ where we assumed that all PB polymers behave like unperturbed, entangled chains. The resulting number density predicts approximately 90 interwoven PB molecules within the volume of one unperturbed chain. In our model of a primitive lattice the polymer volume per unit cell is (*g* + *p*)^3^ − *g* ^3^ and therefore the area density Σ_*PB*_ of carboxyl groups of our bifunctionalized PB on the surface of the nanoparticles is ${{\rm{\Sigma }}}_{PB}=\frac{{n}_{PB}({(g+p)}^{3}-{g}^{3})}{6{g}^{2}}=0.21\,n{m}^{-2}$. This is identical with the grafting density of PB on the magnetite nanoparticles. Compared with our results of an oleic acid/iron oxide nanocomposite, this area density should lead to a roughly 30% higher strength, if it is considered that the carboxyl groups control the strength.

## Conclusions

This work demonstrates the fabrication of nanocomposites via combination of globular iron oxide nanoparticles with a α,ω-polybutadiene dicarboxylic acid. The chemical crosslinking of the elastomer tethered to iron oxide nanoparticles at the moderate processing temperature of 145 °C, which leads to unexpectedly stiff and strong nanocomposites since the polymer volume fraction is approximately 40%. It was found that the elastic modulus and the hardness of the nanocomposites attains values in the range of approximately 12–20 GPa and 15–18 GPa, respectively, although the polymer volume fraction is up to 40%. Our results strongly indicate that the crosslinking and filler percolation yields high elastic moduli. This work also confirms the hypothesis that for nanometer distances between the nanoparticles, it is not necessary to use nanoparticles of high aspect ratio in order to achieve high elastic moduli and strength. Future work should be directed towards a systematic change of the volume fraction of the α,ω-polybutadiene dicarboxylic acid to determine the influence of the size effect for a transition from hard brittle to soft viscoelastic or quasi-ductile behavior of the nanocomposite. Additionally, further studies could be done to investigate the influence of iron oxide nanoparticles to the crosslinking reaction of polybutadiene *via* varying the filling degree of the composites as well as the crosslinking temperature.

## Methods

All applied methods are described in the supplementary information in more detail. The following is a brief summary.

### Polymer and magnetite nanoparticle synthesis

The polymer used in this study is the commercially available low molecular weight Poly bd® R-45HTLO hydroxyl terminated polybutadiene resin with a number average molecular weight of 2800 g ∙ mol^−1^, a polydispersity index of 2.5, and a degree of end-functionalization of 84%. The hydroxyl functional end groups gave opportunity for chemical modification in order to achieve carboxyl functional end groups^10, 11^. Therefore, α,ω-polybutadiene diol initially reacted with an excess of succinic anhydride in presence of dimethylamine pyridine for 48 h at room temperature in dry chloroform. The end-functionalized polymer was precipitated in cold methanol and washed thoroughly with distilled water.

The iron oxide nanoparticles were synthesized according to a scale up of the protocol of Yu *et al*.^38^ Briefly, a mixture of 12.40 g FeO(OH) (0.139 mol), 294.0 g oleic acid (1.04 mol) and 500 g 1-octadecene was heated under nitrogen at 320 °C for 2 hours. The size of the particles was controlled by varying the molar ratio of the precursor and the stabilized as well as of the reaction reaction time. For the exchange of the oleic acid to the polybutadiene iron oxide particles were mixed in a molar ratio of oleic acid to α,ω-polybutadiene dicarboxylic acid of 1:400. Details on the synthesis and the exchange of the ligands are given in the Supporting Information.

### Ligand exchange: Oleic acid/polybutadiene

To exchange the oleic acid of the magnetite particles by PB, the polymer was dissolved in tetrahydrofuran (THF) and mixed with a dispersion of the iron oxide particles using a molar ratio of oleic acid to PB acid of 1:400. The mixture was stirred for 4 hours. Afterwards the particles were separated with ultracentrifugation and the supernatant solution containing any unlinked polymer was removed. The particles were dispersed again in THF and precipitated by adding acetone. The whole procedure - including subsequent addition of PB - was repeated twice. ATR-IR at both particle systems clearly indicates a successful exchange of the ligand.

### ^1^H-NMR

Nuclear magnetic resonance (^1^H-NMR) was accomplished with the *Avance 500* spectrometer (*Bruker Biospin*, *Rheinstetten*, *Germany*), equipped with a 500 MHz magnet and a triple resonance inverse (TXI) probe. The experiment was carried out at room temperature with deuterated chloroform as solvent and tetramethylsilane as internal standard.

### ATR-IR

Fourier transform infrared spectroscopy was performed with a *Bruker Alpha* FT-IR spectrometer (*Bruker Optik*, *Ettlingen*, *Germany*) in the attenuated total reflectance mode (ATR), equipped with an ATR-diamond crystal, in a spectral range of 400–4000 cm^−1^ with a resolution of 2 cm^−1^ and 64 scans.

### DSC

Thermal analysis was accomplished via differential scanning calorimetry measurements using the calorimeter DSC 1 (*Mettler-Toledo*, *Greifensee*, *Switzerland*). The temperature range of the experiments was −120 °C up to −50 °C under a nitrogen atmosphere.

### Helium pycnometry

The measurements were performed on 50–100 mg coarse powder of the nanohybrid material with a micromeritics Accu Pyc II 1340 in a 1 cm² measuring cell. After 100 purge cycles, 70 measuring cycles with a fill pressure of 134 kPa and an equilibration limit of 0.07 kPa ∙ min^−1^ follow.

### TEM, HRTEM, and STEM

The size measurements of the nanoparticles were carried out on a JEOL JEM 1011 at 100 kV and on a JEOL JEM 2200 FS at 200 kV equipped with two CEOS Cs correctors (CETCOR, CESOR) and a Gatan Scan 4 K Ultra Scan 1000 camera. The synthesized nanoparticles were diluted in toluene and dropped on a carbon coated 400 mesh TEM grid. The excess of solvent was removed with a filter paper and the grid was dried under air. The nanocomposite was crushed into a fine powder, which was dispersed in methanol and dropped on a lacey carbon coated 400 mesh TEM copper grid and subsequently dried.

Imaging and SAED was performed with a FEI Talos F200X (X-FEG 200 kV) with a probe current of 50 pA for STEM-Imaging.

## Electronic supplementary material

Supplementary Information

## References

[1] Gao H, Ji B, Jäger IL, Arzt E, Fratzl P (2003). Materials become insensitive to flaws at nanoscale: lessons from nature. Proceedings of the National Academy of Sciences.

[2] Walther A, Berglund L, Ikkala O (2010). Biomimetic, Large-Area, Layered Composites with Superior Properties. European Cells and Materials.

[3] Sellinger A (1998). Continuous self-assembly of organic–inorganic nanocomposite coatings that mimic nacre. Nature.

[4] Podsiadlo P (2007). Ultrastrong and stiff layered polymer nanocomposites. Science.

[5] Bonderer LJ, Studart AR, Gauckler LJ (2008). Bioinspired design and assembly of platelet reinforced polymer films. Science.

[6] Podsiadlo P (2010). The role of order, nanocrystal size, and capping ligands in the collective mechanical response of three-dimensional nanocrystal solids. Journal of the American Chemical Society.

[7] Das, P. *et al*. Nacre-mimetics with synthetic nanoclays up to ultrahigh aspect ratios. *Nature Communications* **6** (2015). 10.1038/ncomms6967 25601360

[8] Dreyer A (2016). Organically linked iron oxide nanoparticle supercrystals with exceptional isotropic mechanical properties. Nature Materials.

[9] Stempflé P, Pantalé O, Rousseau M, Lopez E, Bourrat X (2010). Mechanical properties of the elemental nanocomponents of nacre structure. Materials Science and Engineering: C.

[10] Georgopanos P, Filiz V, Handge UA, Abetz V (2016). Chemical Modification, Thermal Characterization and Dielectric Spectroscopy of Polystyrene‐block‐Polyisoprene Diblock Copolymers. Macromolecular Chemistry and Physics.

[11] Schmidtke C (2013). Amphiphilic, cross-linkable diblock copolymers for multifunctionalized nanoparticles as biological probes. Nanoscale.

[12] Yu S, Chow GM (2004). Carboxyl group (–CO_2_H) functionalized ferrimagnetic iron oxide nanoparticles for potential bio-applications. Journal of Materials Chemistry.

[13] Fischer S, Salcher A, Kornowski A, Weller H, Förster S (2011). Completely miscible nanocomposites. Angewandte Chemie International Edition.

[14] Mitchell GP, Mirkin CA, Letsinger RL (1999). Programmed assembly of DNA functionalized quantum dots. Journal of the American Chemical Society.

[15] Wei H (2011). Compact zwitterion-coated iron oxide nanoparticles for biological applications. Nano Letters.

[16] Korpany KV, Majewski DD, Chiu CT, Cross SN, Blum AS (2017). Iron Oxide Surface Chemistry: Effect of Chemical Structure on Binding in Benzoic Acid and Catechol Derivatives. Langmuir.

[17] Ling D, Hyeon T (2013). Chemical design of biocompatible iron oxide nanoparticles for medical applications. Small.

[18] Raue M (2014). Investigation of historical hard rubber ornaments of Charles Goodyear. Macromolecular Chemistry and Physics.

[19] Liaqat F (2015). Ultrastrong composites from dopamine modified-polymer-infiltrated colloidal crystals. Materials Horizons.

[20] Han L, Wang L, Song J, Boyce MC, Ortiz C (2011). Direct quantification of the mechanical anisotropy and fracture of an individual exoskeleton layer via uniaxial compression of micropillars. Nano Letters.

[21] Sneddon IN (1965). The relation between load and penetration in the axisymmetric Boussinesq problem for a punch of arbitrary profile. International Journal of Engineering Science.

[22] Choperena A, Painter P (2009). Hydrogen bonding in polymers: effect of temperature on the OH stretching bands of poly (vinylphenol). Macromolecules.

[23] de Lucca Freitas L, Auschra C, Abetz V, Stadler R (1991). Hydrogen bonds in unpolar matrix—Comparison of complexation in polymeric and low molecular-weight systems. Colloid & Polymer Science.

[24] Du N, Dal-Cin MM, Robertson GP, Guiver MD (2012). Decarboxylation-induced cross-linking of polymers of intrinsic microporosity (PIMs) for membrane gas separation. Macromolecules.

[25] Kratochvil AM, Koros WJ (2008). Decarboxylation-induced cross-linking of a polyimide for enhanced CO2 plasticization resistance. Macromolecules.

[26] Liu C, He J, Van Ruymbeke E, Keunings R, Bailly C (2006). Evaluation of different methods for the determination of the plateau modulus and the entanglement molecular weight. Polymer.

[27] Meltzer T, Dermody W, Tobolsky A (1965). Fraction of effective sulfur crosslinking in polybutadiene rubber vulcanizates. Journal of Applied Polymer Science.

[28] Strobl GR. *The Physics of Polymers*. Springer (2007).

[29] Morrison N, Porter M (1984). Temperature effects on the stability of intermediates and crosslinks in sulfur vulcanization. Rubber Chemistry and Technology.

[30] Scott J (1942). The plastic-elastic behaviour of ebonite. Transactions of the Faraday Society.

[31] Handge UA, Wolff MF, Abetz V, Heinrich S (2016). Viscoelastic and dielectric properties of composites of poly (vinyl butyral) and alumina particles with a high filling degree. Polymer.

[32] Georgopanos P (2017). Improvement of mechanical properties by a polydopamine interface in highly filled hierarchical composites of titanium dioxide particles and poly(vinyl butyral). Composites Science and Technology.

[33] Reichmann HJ, Jacobsen SD (2004). High-pressure elasticity of a natural magnetite crystal. American Mineralogist.

[34] Treloar L (1973). The elasticity and related properties of rubbers. Reports on Progress in Physics.

[35] Mansilla M (2013). Evolution of the free volume and glass transition temperature with the degree of cure of polybutadiene rubbers. Polymer Testing.

[36] Fetters, L., Lohse, D. & Colby, R. Chain dimensions and entanglement spacings. In: *Physical Properties of Polymers Handbook* (eds). Springer (2007).

[37] Koch M, Sommer J-U, Blumen A (1997). Polymer chains tethered to impenetrable interfaces: Broadening of relaxation spectra. The Journal of Chemical Physics.

[38] William, W. Y., Falkner, J. C., Yavuz, C. T. & Colvin, V. L. Synthesis of monodisperse iron oxide nanocrystals by thermal decomposition of iron carboxylate salts. *Chemical Communications* 2306–2307 (2004). 10.1039/b409601k 15489993

