# Supplementary material for: Exceptionally strong, stiff and hard hybrid material based on an elastomer and isotropically shaped ceramic nanoparticles
Source: Sci Rep. 2017 Aug 4;7:7314. doi: 10.1038/s41598-017-07521-0 (PMC5544721; doi:10.1038/s41598-017-07521-0)
Supplement: Supplementary file 1 — Supplementary Information [file 41598_2017_7521_MOESM1_ESM.doc]

Supplementary Information

**Exceptionally strong, stiff and hard hybrid material based on an elastomer and isotropically shaped ceramic nanoparticles**

Prokopios Georgopanos,^1^ Gerold A. Schneider,^2^ Axel Dreyer,^2^ Ulrich A. Handge,^1^ Volkan Filiz,^1^ Artur Feld,^3^ Ezgi D. Yilmaz,^2^ Tobias Krekeler,^4^ Martin Ritter,^4^ Horst Weller,^3^ and Volker Abetz^1,3^

^1^ Institute of Polymer Research, Helmholtz-Zentrum Geesthacht, Max-Planck-Strasse 1, D-21502 Geesthacht, Germany

^2^ Institute of Advanced Ceramics, Hamburg University of Technology, Denickestrasse 15, D-21073 Hamburg, Germany

^3^ Institute of Physical Chemistry, Hamburg University, Martin-Luther-King-Platz 6, D-20146 Hamburg, Germany

^4^ Electron Microscopy Unit, Hamburg University of Technology, Eißendorferstraße 42, D-21073 Hamburg, Germany

Email: [g.schneider@tuhh.de](mailto:g.schneider@tuhh.de), [volker.abetz@hzg.de](mailto:volker.abetz@hzg.de)

**A Synthesis and characterization of α,ω-polybutadiene dicarboxylic acid**

The Poly bd® R-45HTLO hydroxyl terminated polybutadiene resin was chemically modified in order to achieve –COOH functional end groups as it is described in previous works.^[1,2]^ Therefore α,ω-polybutadiene diol initially reacted with four fold excess of succinic anhydride (Sigma-Aldrich, Schnelldorf, 99%) in presence of dimethylamine pyridine (Sigma-Aldrich, Schnelldorf, 99%) for 48 h at room temperature. The solvent used was dry chloroform. The end-functionalized polymer was precipitated in cold methanol and washed thoroughly with distilled water. The modified polymer was chemically characterized by nuclear magnetic resonance spectroscopy (^1^H-NMR), Fourier transform infrared spectroscopy (FT-IR) and differential scanning calorimetry (DSC). Nuclear magnetic resonance (^1^H-NMR) was accomplished with the *Avance 500* spectrometer (*Bruker Biospin, Rheinstetten, Germany*), equipped with a 500 MHz magnet and a triple resonance inverse (TXI) probe. The experiment was carried out at room temperature with deuterated chloroform as solvent and tetramethylsilane as internal standard. Fourier transform infrared spectroscopy was performed with a *Bruker Alpha* FT-IR spectrometer (*Bruker Optik, Ettlingen, Germany*) in the attenuated total reflectance mode (ATR), equipped with an ATR-diamond crystal, in a spectral range of 400 – 4000 cm^‑1^ with a resolution of 2 cm^−1^ and 64 scans. The viscous polymer was measured directly on the diamond crystal. Thermal analysis was accomplished via differential scanning calorimetry measurements using the calorimeter DSC 1 (*Mettler-Toledo, Greifensee, Switzerland*). The temperature range of the experiments was −120 °C up to -50 °C under a nitrogen atmosphere. A heating and cooling rate of 10 K∙min^-1^ was used. The second heating interval was analyzed for the determination of the glass transition temperature. Based on the analysis of the DSC experiments, a glass transition temperature was detected at -80 °C for the α,ω-polybutadiene diol and at -78 ^o^C for the α,ω−polybutadiene dicarboxylic acid in agreement with what is expected for the blend of a low molecular weight polybutadiene of 1,4 and 1,2 microstructures.^[3]^ From the ^1^H-NMR characterization, the chemical shifts attributed to the protons near the double bonds of the different polybutadiene microstructures can be seen in the interval from approximately 4 up to 6 ppm. The characteristic chemical shifts from the protons near the carboxyl groups appeared at approximately 2.6 and 3.0 ppm. The proton of the carboxyl group is not visible due to the very fast exchange in CHCl_3_. Additional characterization through FT-IR measurements verified the addition of carboxyl groups at the end of the polymer chain. The characteristic stretching vibration of the C=O bond was found at approximately 1700 cm^-1^.

**B Synthesis and characterization of the iron oxide nanoparticles**

Magnetite nanocrystals were synthesized in accordance to Yu et al.^[4]^. Briefly, a mixture of 12.40 g FeO(OH) (0.139 mol), 294.0 g oleic acid (1.04 mol) and 500 g 1-octadecene was heated under nitrogen at 320 °C for 2 hours. After synthesis the particles were precipitated by adding acetone, centrifuged and stored in toluene. To exchange the oleic acid of the original particles by α,ω-polybutadiene dicarboxylic acid the polymer was dissolved in tetrahydrofuran (THF) and mixed with a dispersion of the iron oxide particles using a molar ratio of oleic acid to α,ω-polybutadiene dicarboxylic acid of 1:400. The mixture was stirred for 4 hours. Afterwards the particles were precipitated by adding acetone. The whole procedure - including subsequent addition of α,ω-polybutadiene dicarboxylic acid - was repeated twice.

To obtain the bulk solid material the particles were precipitated with acetone and redispersed in tetrahydrofuran by ultrasonification and subsequently poured into a sealed die resulting in sedimentation and self-assembly.

The aggregation of the nanoparticles was induced by slow evaporation of solvent within several days at room temperature. Thereafter, the remaining sediment was dried under vacuum at room temperature and uniaxially pressed in air at 145 ± 5 °C with a pressure of 260 MPa for 7 hours.

To crosslink the PB coated particles by sulfur (vulcanization), 493 mg of these particles were dispersed in 3 mL of a 0.74 weight-% THF sulfur solution before pouring into the die.

*Transmission Electron Microscopy*

To obtain a sufficiently thin specimen of the pressed nanocomposites, a small amount of the bulk solid was ground in a mortar and dispersed in Methanol. One drop of this dispersion was applied to a copper TEM grid (400 Mesh covered with lacey carbon) and thoroughly dried in vacuum.

SAED (selected area electron diffraction) was performed on a 10 x 10 µm chunk of the pressed nanocomposite, leading to a diffraction pattern corresponding to polycrystalline, nanoscale magnetite (see Figure SI 1).

Imaging is done via HAADF (high angle annular dark field) imaging. This STEM (scanning transmission electron microscopy) technique uses incoherently scattered electrons, resulting in image intensities solely dependent to the specimen thickness and the atomic number of the containing elements, reducing any other image contrast formation (e.g. bragg contrast). For STEM-Imaging a FEI Talos F200X (X-FEG) with a probe current of 50 pA has been used.

**
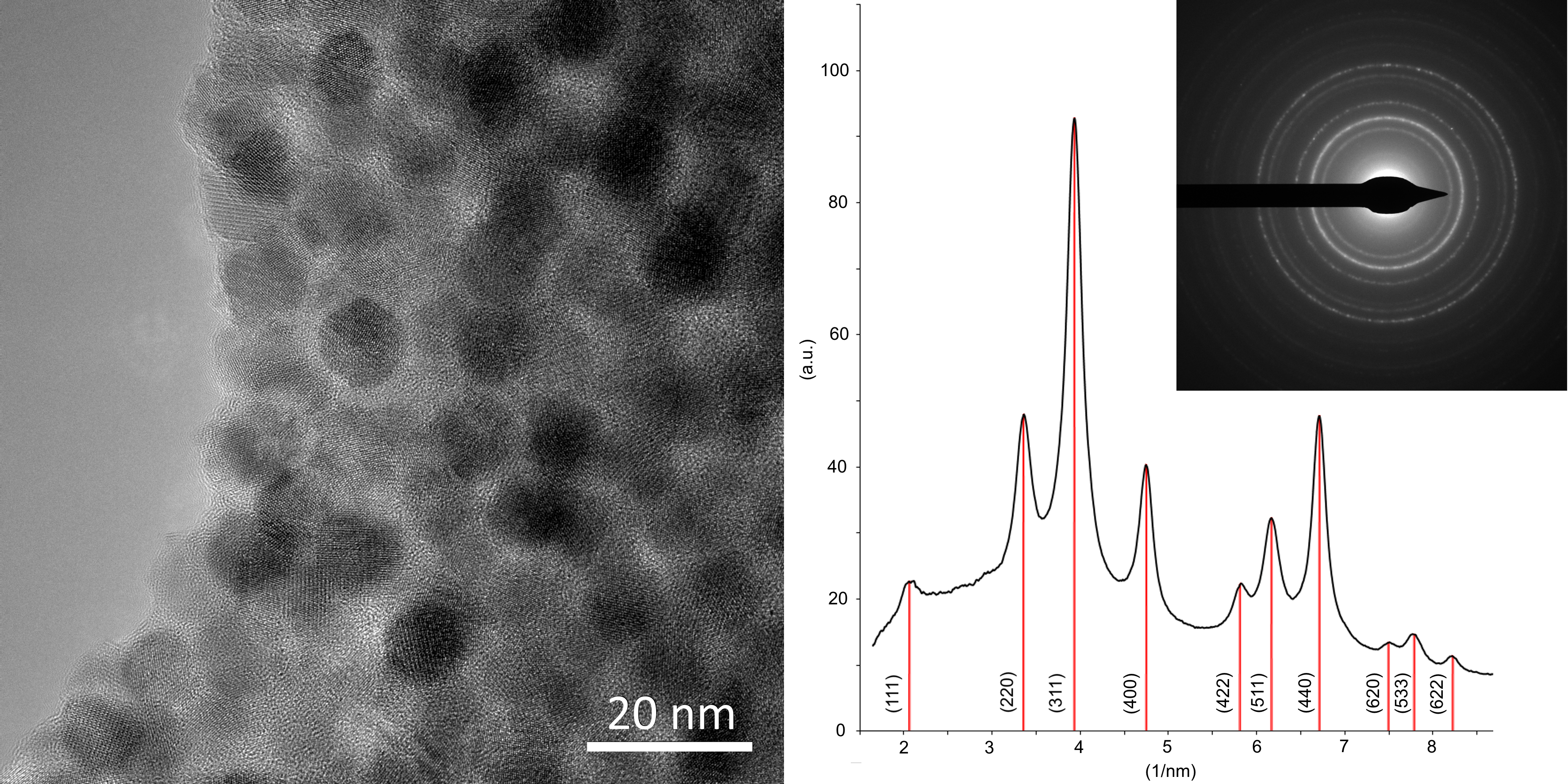
**

**Figure SI 1.** SAED measurements of the hot-pressed nanocomposites (pictured on the left) with the corresponding, background corrected rotation profile (black line) of the electron diffraction pattern (inset). The measured radii of the diffraction rings correspond to the lattice plane spacings of magnetite (Fe_3_O_4_) (COD No. 1010369) (highlighted in red and indexed).

*IR spectroscopy*

The IR spectroscopy shows the success of the exchange of the oleic acid by α,ω-polybutadiene dicarboxylic acid. The samples were investigated as a film on a diamond ATR crystal Brucker Platinum Alpha. The increase of the C=C-valence vibration at 3010 cm^-1^ compared to the COOH- and COO^-^-deformation vibration in the Figure SI 2 indicates the α,ω-polybutadiene dicarboxylic acid coverage.

**Figure SI 2.** Attenuated total reflectance infrared spectra of the pressed particle systems iron oxide coated with oleic acid (Fe_3_O_4_@oleic acid), α,ω-polybutadiene dicarboxylic acid (Fe_3_O_4_@PB(COOH)_2_) and α,ω-polybutadiene dicarboxylic acid with sulfur (Fe_3_O_4_@PB(COOH)_2_+S).

*Thermogravimetric analysis*

Thermogravimetric analysis (TGA) using a Mettler Toledo 1 STARe System at the nanocomposites show a similar stepwise decrease of the organic mass fraction during heating-up with rate of 1 K∙min^-1^ under a nitrogen atmosphere (Figure SI 3).

**Figure SI 3.** TGA measurement of the hot-pressed nanocomposite at a heating rate of 1 K∙min^-1^ in a N_2_-flow shows the successive desorption of organic matter. Its negative derivative shows maxima in the desorption rate between 100 to 450 °C and 600 to 800 °C.

*Influence of thermal treatment on mechanical properties*

A subsequent thermal treatment at 145 °C of the iron oxide/α,ω-polybutadiene dicarboxylic acid/sulfur nanocomposite over 35 hours reveals an alternate increase and decrease in mechanical properties between mean values of 12 GPa and 20 GPa in the elastic modulus and between 15 GPa and 18 GPa in the hardness. Overall there is no clear trend for a substantial increase in the mechanical properties (Figure SI 4).

**Figure SI 4.** Development of the mechanical properties of iron oxide/α,ω−polybutadiene dicarboxylic acid/sulfur nanocomposite after during thermal treatment over 35 hours.

*Energy-dispersive X-ray analysis*

The spatial distribution of the sulfur in the nanocomposite was measured by energy-dispersive X-ray analysis (EDX) using a AZtec EDX-System from Oxford Instruments with a fast 80 mm² X-max SDD-detector. All measurements were performed with a working distance of 8.5 mm and excitation energy of 20 kV. At magnification 1000 three areas at the top- and bottom side of the sample were analyzed. The following Table SI 1 contains the determined sulfur contents. The sulfur content exhibits an average gradient of 0.65±0.12 weight-%∙cm^-1^ over the pellet height from 4.32 ± 0.56 weight-% on the top side to 3.74 ± 0.33 weight-% on the bottom side. This is a result of different onsets of the precipitation of the particles and the sulfur during the evaporation of the common solvent. First the particles start to aggregate and sediment. Later, the sulfur starts to precipitate when crossing the solubility concentration limit.

**Table SI 1.** Sulfur mass fraction at different areas of a nanocomposite sample iron oxide/α,ω-polybutadiene dicarboxylic acid/sulfur measured by EDX.

| Sample area | Sulfur mass fraction / weight-% | | | Aver. sulfur mass fraction / weight-% | Standard deviation / weight-% |
| --- | --- | --- | --- | --- | --- |
| Top side | 4.95 | 4.15 | 3.88 | 4.32 | +/-0.56 |
| Bottom side | 3.04 | 3.67 | 3.21 | 3.31 | +/-0.33 |
| Side | 2.81 | 2.86 | 2.26 | 2.65 | +/-0.33 |
|  | total sulfur mass fraction / weight-% | | | 3.43 | +/-0.82 |

**C Mechanical characterization of the nanocomposites**

*Nanoindentation.* They were performed with the Agilent Nano Indenter G200 system using the continuous stiffness measurement option (CSM) with a constant strain target of 0.05 s^-1^. The maximum indentation depth was set to 2000 nm.

*Micromechanical tests.* The microbending and microcompression tests were conducted in the nanoindenter equipped with a Berkovich tip and a flat-ended punch being 10 µm in diameter, respectively. Force (*F*), displacement (*δ*), and stiffness (*S*) data were recorded continuously as a function of indentation displacement. The formulae employed to evaluate the stress–strain (*σ–*
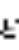
) curves and the elastic modulus (*E*) of the specimens are as follows:

*Microbending*


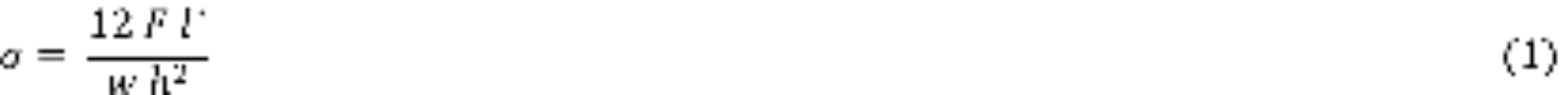


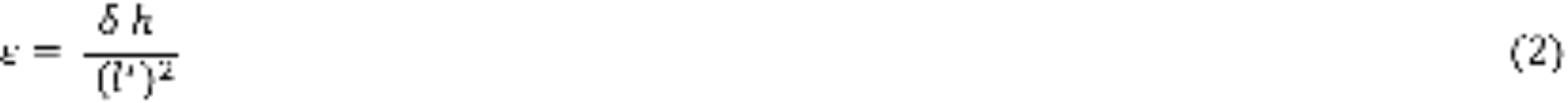


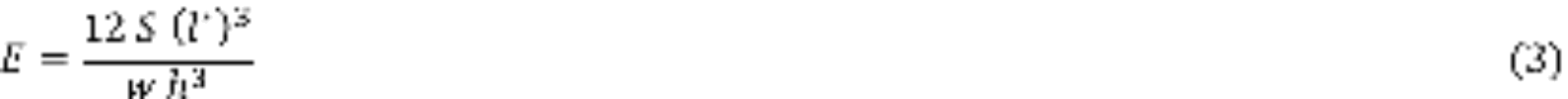


where *l** is the loaded length of the cantilever, *w* is the width and *h* is the height of the cantilevers ^[5]^.

*Microcompression*


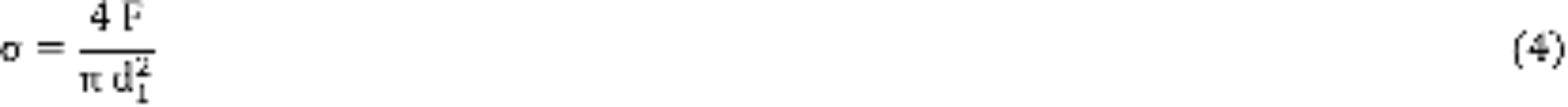


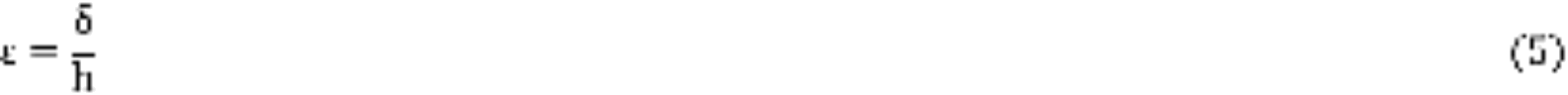


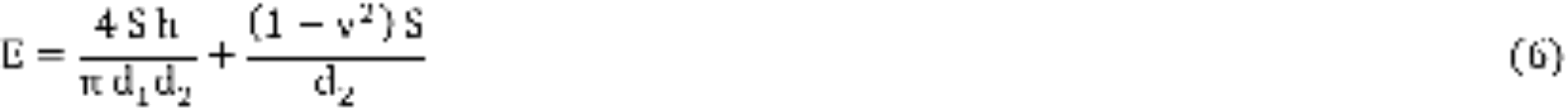


where *d_1_*, *d_2_, h* are the top diameter, bottom diameter and the height of the pillars, respectively.
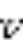
 is the Possion`s ratio taken as 0.25. (It is known that the elastic modulus formula employed in microcompression tests has to be corrected due to the geometrical constraints. We adapted the formula introduced in Han et al. ^[6]^ based on Sneddon et al. ^[7]^.

The images of the representative specimens prior the measurements are illustrated in Figure SI 5. The geometrical data of the tested specimens are given in Tables SI 2 and SI 3. A more detailed explanation of the conduction of the tests and evaluation of the mechanical data is given in by Dreyer et al.^[8]^


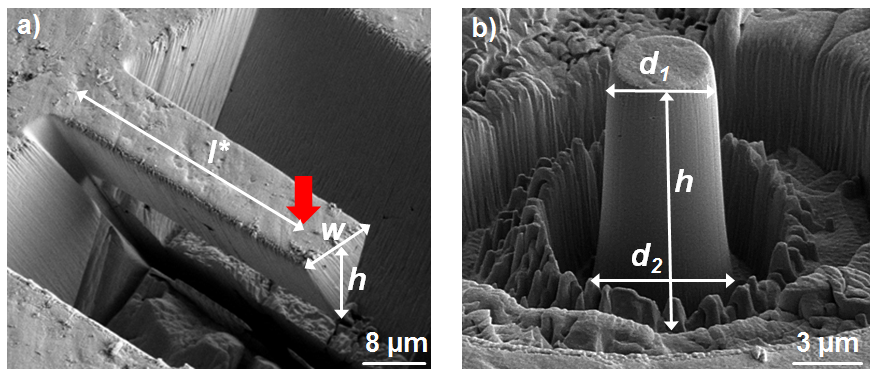


**Figure SI 5.** In (a) a representative microcantilever fabricated in this study is demonstrated. *l** denotes the loaded cantilever length measured as the distance between the cantilever base and the indenter loading point marked with the red arrow. *w* and *h* are the width and height of the cantilever, respectively. A representative micropillar is shown in (b). *d_1_*, *d_2_*and *h* denote the top diameter, the bottom diameter and the height of the pillars.

The micromechanical test leads to the force-displacement curves presented in Figure SI 6. Calculations according to equation (1 to 6) deliver results for the elastic modulus and strength collected in Table SI 2 for bending test and Table SI 3 for compression tests.

**
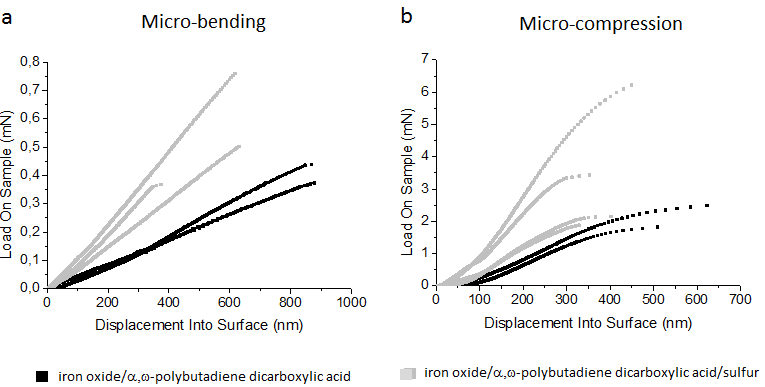
**

**Figure SI 6.** Force-displacement curves obtained under micro-bending (a) and micro-compression (b); and stress-strain curves obtained under micro-bending (c) and micro-compression (d) are plotted. Light grey and black colors represent the cross-linked and non-cross-linked sample groups, respectively.

**Table SI 2.** The dimensions and mechanical properties of the micro-bending specimens

| **Specimens** | **Width**  ***w***  **(µm)** | **Height**  ***h***  **(µm)** | **Length**  ***l****  **(µm)** | **Failure strength***  ***σ***  **(MPa)** | **Elastic modulus**  ***E***  **(GPa)** |
| --- | --- | --- | --- | --- | --- |
| *Cross-linked specimens* | | | | | |
| 1 | 8.4 | 9.2 | 22,0 | 281 | 32 |
| 2 | 8.3 | 9.2 | 20,0 | 173 | 16 |
| 3 | 8.9 | 9.6 | 20,0 | 108 | 20 |
| mean ± s.d. |  | | | 184 ± 87 | 23 ± 8 |
| *Non-crosslinked specimens* | | | | | |
| 1 | 7.8 | 8.8 | 21.0 | 184 | 16 |
| 2 | 7.8 | 8.3 | 24.0 | 198 | 23 |
| mean ± s.d. |  | | | 191 ± 10 | 20 ± 5 |

*Strength values are calculated by the maximum force attained during the measurements.

**Table SI 3.** The dimensions and mechanical properties of the microcompression specimens

| **Specimens** | **Top diameter**  ***d_1_***  **(µm)** | **Bottom diameter**  ***d_2_***  **(µm)** | **Height**  ***h***  **(µm)** | **Failure**  **strength***  ***σ***  **(MPa)** | **Elastic modulus**  ***E***  **(GPa)** |
| --- | --- | --- | --- | --- | --- |
| *Cross-linked specimens* | | | | | |
| 1 | 3.1 | 3.9 | 9.5 | 842 | 10 |
| 2 | 3.1 | 3.9 | 9.9 | 459 | 8 |
| 3 | 2.9 | 3.9 | 9.9 | 316 | 7 |
| 4 | 2.9 | 3.9 | 9.9 | 287 | 6 |
| mean ± s.d. |  | | | 476 ± 255 | 8 ± 2 |
| *Non-crosslinked specimens* | | | | | |
| 1 | 2.9 | 3.9 | 11.9 | 290 | 13 |
| 2 | 2.9 | 3.8 | 11.6 | 447 | 15 |
| mean ± s.d. |  | | | 369 ± 111 | 14 ± 1 |

*Strength values are calculated by the maximum force attained during the measurements.]

**D Rheological measurements**

These experiments were carried out using the rotational rheometer MCR 502 (Anton Paar GmbH, Graz, Austria). Rheological tests were accomplished using α,ω-polybutadiene dicarboxylic acid and α,ω-polybutadiene dicarboxylic acid/sulfur at a temperature of 145 ^o^C.

The preparation of the samples started dissolving 0.5 g polymer in 2 mL THF. For the samples containing sulfur, approximately 15 weight-% or 0.08 g of sulfur (Sigma Aldrich, Schnelldorf, 99.8%) was dissolved with the use of an ultrasonic bath in approximately 1 mL THF and afterwards added to the polymer solution. The solution was homogenized with the use of an ultrasonic bath and a shaker. Then the samples were slowly dried over a period of 6 days and subsequently vacuum dried for at least 1 hour before the rheological investigations in order to remove residues of the solvent. A parallel plates geometry with disposable tools of 25 mm diameter was used. The gap for the measurements was set to approximately 0.2 mm. The temperature for the rheological measurements was chosen following the parameters for preparation of the nanocomposites and was equal to 145 °C.

Several rheological experiments were performed in order to analyze the crosslinking kinetics. Amplitude sweep experiments at an angular frequency of 10 rad s^-1^ were carried out with a shear amplitude γ_0_ varying in the range from 1 to 10 % in order to determine the linear viscoelastic range. Frequency sweep experiments were performed before and after the in-situ vulcanization - crosslinking of the polymer in the rheometer. The frequency range was ω = 100 – 0.1 rad s^-1^ and the shear amplitude 5%. In order to verify that the crosslinked structure does not break up at higher deformations, frequency sweeps with shear amplitudes of 5% and 20% were also carried out. Time sweep experiments at 145 °C were accomplished in order to monitor the kinetics of the vulcanization-crosslinking of the polymer during the measurement. The angular frequency was 0.1 rad s^-1^ and the shear amplitude 5%. The vulcanization time for the test on the sample with sulfur in the absence of oxygen was estimated by the time *t*_90_ corresponding to the value where the storage modulus exhibits 90% of its equilibrium (saturation) value ^[9]^. Rheological experiments under nitrogen as well as under air atmosphere were performed in order to study the influence of oxygen on the crosslinking kinetics. In both cases the time sweep experiments on oleic acid at 145 ^o^C do not indicate a crosslinking reaction because of dynamic moduli lower than 10^-1^ Pa.

Additional frequency sweep experiments reveal that the moduli after vulcanization do not depend on shear amplitude which was varied from 5% to 50% in different tests. In Figure SI 7 the frequency sweeps in the range of ω = 100 - 0.1 rad/s of the vulcanized α,ω-polybutadiene dicarboxylic acid with 15% sulfur are presented. As indicated before the crosslinked structure does not break up at higher deformations and both dynamic moduli are not influenced by the increase of the shear amplitude.


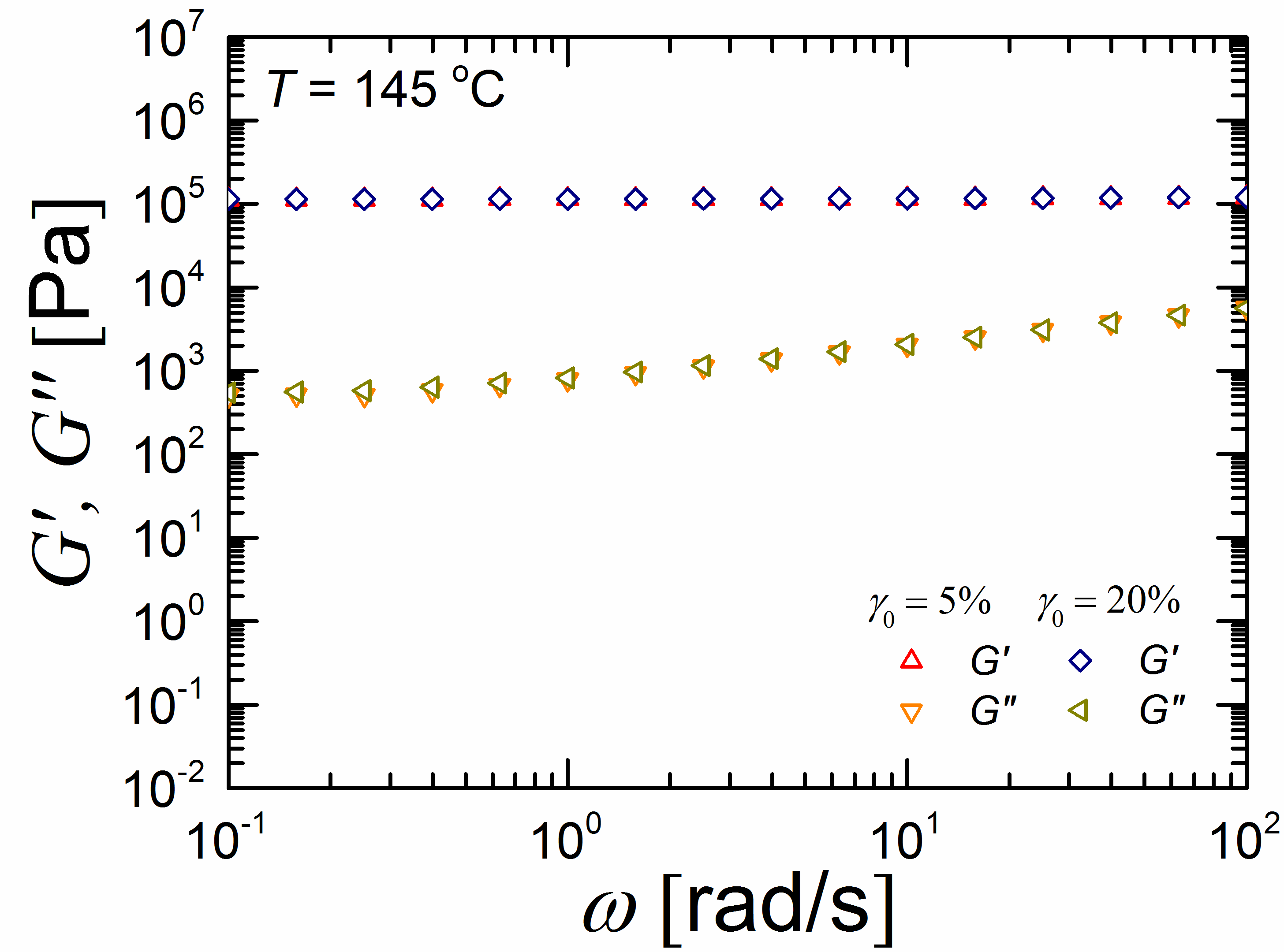


**Figure SI 7.** Frequency sweeps data for shear amplitude of γ_0_ = 5% and γ_0_ = 20% the vulcanized α,ω-polybutadiene dicarboxylic acid with 15% sulfur at 145 ^o^C under nitrogen.

**References**

[1] Georgopanos P, Filiz V, Handge UA, Abetz V. Chemical Modification, Thermal Characterization and Dielectric Spectroscopy of Polystyrene‐block‐Polyisoprene Diblock Copolymers. *Macromolecular Chemistry and Physics* **217**, 1293-1304 (2016).

[2] Schmidtke C*, et al.* Amphiphilic, cross-linkable diblock copolymers for multifunctionalized nanoparticles as biological probes. *Nanoscale* **5**, 7433-7444 (2013).

[3] Röthemeyer F, Sommer F, *Kautschuktechnologie: Werkstoffe-Verarbeitung-Produkte*, Carl Hanser Verlag GmbH Co KG, (2013).

[4] Yu S, Chow GM. Carboxyl group (–CO_2_H) functionalized ferrimagnetic iron oxide nanoparticles for potential bio-applications. *Journal of Materials Chemistry* **14**, 2781-2786 (2004).

[5] Dubbel H., Beitz W., Küttner K.-H., *Dubbel: Taschenbuch für den Maschinenbau*, Springer-Verlag, (2013)

[6] Han L, Wang L, Song J, Boyce MC, Ortiz C. Direct quantification of the mechanical anisotropy and fracture of an individual exoskeleton layer via uniaxial compression of micropillars. *Nano Letters* **11**, 3868-3874 (2011).

[7] Sneddon IN. The relation between load and penetration in the axisymmetric Boussinesq problem for a punch of arbitrary profile. *International Journal of Engineering Science* **3**, 47-57 (1965).

[8] Dreyer A*, et al.* Organically linked iron oxide nanoparticle supercrystals with exceptional isotropic mechanical properties. *Nature Materials* **15**, 522-528 (2016).

[9] Raue M*, et al.* Investigation of historical hard rubber ornaments of Charles Goodyear. *Macromolecular Chemistry and Physics* **215**, 245-254 (2014).
